# Discordance between apolipoprotein B or non-HDL-cholesterol and LDL-cholesterol in middle-aged and elderly Chinese patients predicts arterial stiffness

**DOI:** 10.1186/s12944-021-01509-6

**Published:** 2021-07-29

**Authors:** Geyue Qu, Zhongying Zhang, Hong Zhu

**Affiliations:** Department of Geriatrics, Xuanwu Hospital, Capital Medical University, Beijing, 100053 China

**Keywords:** Arterial stiffness, Discordance, Non-high-density lipoprotein cholesterol, Apolipoprotein B, Low-density lipoprotein cholesterol

## Abstract

**Background:**

Discordance of lipid parameters is closely associated with residual cardiovascular risk. This study investigated the discordance between non-high-density lipoprotein cholesterol (non-HDL-C) or apolipoprotein B (apoB) and low-density lipoprotein cholesterol (LDL-C), and assessed arterial stiffness risk.

**Methods:**

This study included a total of 402 middle-aged and elderly Northern Chinese individuals whose brachial-ankle pulse wave conduction velocity (baPWV), and clinical and biochemical data were measured. Arterial stiffness was defined by inclusion in the upper quartile of the baPWV. All participants were divided into four mutually exclusive concordance/discordance groups based on the lipid goal for high-risk populations, according to the 2019 European Society of Cardiology / European Atherosclerosis Society guidelines. Discordance was defined as LDL-C ≥ 1.81 mmol/L with non-HDL-C <  2.59 mmol/L, or apoB < 0.80 mmol/L, or vice versa.

**Results:**

The mean age of the participants was 65.9 ± 13.0 years; 59.5% of the participants were male. The mean LDL-C was 2.41 ± 0.81 mmol/L, non-HDL-C: 3.06 ± 0.94 mmol/L, and apoB: 0.84 ± 0.21 mmol/L. LDL-C was observed to be discordant with non-HDL-C (20.1%) and apoB (30.8%). When stratified according to LDL-C levels, the baPWV was greater in those patients with higher non-HDL-C or apoB levels. In the adjusted logistic regression model, low LDL-C and high non-HDL-C or apoB discordance were also associated with the risk of arterial stiffness (OR: 13.412 and OR: 13.054, respectively).

**Conclusions:**

There was discordance between LDL-C and non-HDL-C, or apoB in middle-aged and elderly Chinese individuals; this was associated with a higher risk of arterial stiffness. Non-HDL-C or apoB levels could be used to identify individuals who may benefit from more comprehensive lipid modification.

## Background

Hyperlipidemia is associated with a higher risk of cardiovascular disease (CVD). Cholesterol control is easily achievable, and constitutes the central aspect of atherosclerotic CVD prevention. Atherosclerotic CVD is linearly associated with low-density lipoprotein cholesterol (LDL-C); therefore, targeting LDL-C is a recommended strategy for reducing cardiovascular risk. However, many individuals, even those with optimal LDL-C levels, experience cardiovascular events or atherosclerosis progression [1, 2]. This phenomenon has been termed as the “residual risk” [3], which cannot be identified by measuring LDL-C. In addition, exclusive targeting of LDL-C is limited by measurement variability. Thus, interest has been increasing in the use of alternative lipid parameters. Many studies [4–6] have shown that in addition to LDL-C, other lipid parameters, such as non-high-density lipoprotein cholesterol (non-HDL-C) or apolipoprotein B (apoB) can also increase the risk of CVD; this is conducive to the assessment and treatment of residual risk, because the major contributing risk factor for residual risk is the difference between the estimated LDL-C value and the actual quantity of circulating atherogenic lipoprotein particles. Non-HDL-C comprises cholesterol carried by all potentially atherogenic lipoprotein particles, including LDL-C, intermediate-density lipoproteins (IDL), very-low-density lipoproteins, remnant lipoproteins, and lipoprotein a (LPa). ApoB represents the number of atherogenic lipoprotein particles mentioned, because each lipoprotein particle contains one molecule of apoB [7]. LDL-C represents the total cholesterol concentration of LDL, IDL, and LPa particles. Cholesterol content within atherogenic lipoprotein particles varies substantially in approximately 10 to 20% of individuals, and the particles are either enriched or alternatively depleted in cholesterol [8]. When the atherogenic lipoprotein particle concentration within a standardised amount of cholesterol is consistent, the cholesterol concentration is considered to be concordant with the number of lipoprotein particles. The cardiovascular risk can then be accurately predicted by LDL-C, non-HDL-C, and apoB. However, when the cholesterol content is higher or lower than the average concentration, the cholesterol concentration is discordant with the number of lipoprotein particles, as is the cardiovascular risk predicted by LDL-C, non-HDL-C, and apoB. Several previous studies have investigated the discordance between LDL-C and non-HDL-C, or apoB [8–10]. The 2019 European Society of Cardiology / European Atherosclerosis Society (ESC/EAS) guidelines for the management of dyslipidemia [11] advocate that non-HDL-C and apoB should be evaluated and considered as secondary targets for lipid control.

According to the well-known cardiovascular events chain proposed by Dzau and Braunwald, the development of CVD is a continuous process, and arterial stiffness is an important intermediate stage in this progression. The brachial-ankle pulse wave conduction velocity (baPWV) is a reliable parameter for screening for arterial stiffness, which is an independent predictor of cardiovascular morbidity and mortality and a valid surrogate endpoint for CVD [12, 13]. Dyslipidemia is considered to be a possible risk factor for arterial stiffness [14–17], although the underlying mechanism is still unclear. Therefore, the target range and clinical value of controlling blood lipids for arterial stiffness are currently unclear. Most existing analyses of lipid discordance focus on the prediction and assessment of cardiovascular risk.

Regarding the question on whether the relationship between arterial stiffness and blood lipids also affected by the discordance of lipid parameters, it is not clear whether discordance in lipid parameters provides additional clinical information on arterial stiffness. Therefore, the purpose of this study was to investigate the discordance between LDL-C and non-HDL-C or apoB in middle-aged and elderly Chinese individuals and assess the arterial stiffness risk among participants in whom these parameters were discordant.

## Methods

### Study population

This is cross-sectional investigation analysed baseline data collected for a prospective cohort study. The participants were middle-aged and elderly Northern Chinese patients, who underwent annual physical examinations at the Xuanwu Hospital of the Capital Medical University between July 2017 and October 2019. The inclusion criteria for the study were as follows [18]: (1) age 45 years or older, (2) underwent a baPWV examination, and (3) had no missing clinical and biochemical data. Patients with secondary hypertension, acute cardiovascular and cerebrovascular disease, severe arrhythmia, abnormal liver function (i.e., aspartate amino transferase (AST) or alanineaminotransferase (ALT) > 100 U/L), abnormal kidney function (estimatedglomerular filtration rate (eGFR) < 60 mL/min/1.73 m^2^), malignant tumours, infections, thyroid dysfunction, mental disease, and peripheral vascular disease (i.e., ankle/brachial systolic blood pressure index (ABI) < 0.9) were excluded in addition to pregnant and lactating women. A total of 402 participants were included in the analysis.

All subjects provided informed consent prior to participation in the study. The study was conducted in accordance with the Declaration of Helsinki, and the protocol was approved by the Ethics Committee of the Xuanwu Hospital, Capital Medical University (approval number: Clinical research review [2018] No. 038).

### Data collection

Information on age, sex, smoking and alcohol consumption, medical history, and current drug use was obtained by trained physicians using standardised questionnaires. Participants who smoked at least one cigarette per day or drank alcohol once a week for at least 6 months were defined as smokers or drinkers, respectively [19]. Trained staff measured all participants’ height in meters, weight in kilograms, and blood pressure in mmHg. The body mass index (BMI) was calculated as kg/m^2^. An electronic sphygmomanometer was used to measure the participants’ blood pressure in a seated position after a 10 min rest. Triplicate measurements were obtained with a break of at least 2 min between readings; the average value was used for the analysis.

Blood samples were collected in the morning after overnight fasting for at least 8 h. Fasting blood glucose (FBG), total cholesterol (TC), triglyceride (TG), high-density lipoprotein cholesterol (HDL-C), LDL-C, apoB, serum creatinine (Scr), uric acid (UA), and homocysteine levels were measured using an automatic biochemical analyser (Olympus Corporation, Hitachi 7600, Japan). Non-HDL-C was calculated as the difference between TC and HDL-C. The eGFR was calculated using the Chronic Kidney Disease Epidemiology Collaboration (CKD-EPI) method. Haemoglobin (HGB) levels were detected using an automatic blood cell analyser (XE-2100, Hisemori Micon Company, Japan).

Hypertension was defined as systolic blood pressure (SBP) ≥140 mmHg and/or diastolic blood pressure (DBP) ≥ 90 mmHg on at least two blood pressure measurements per visit for at least two visits, and/or prescription of any antihypertensive medication. Diabetes mellitus (DM) was defined as plasma glucose levels of≥7.0 mmol/L for at least two measurements, glycosylated haemoglobin (HbA1C) ≥6.5%, or prescription of any antidiabetic medication.

The baPWV was measured using an oscillometer-based device (BP-203RPE III; Colin-Omron, Co., Ltd., Tokyo, Japan). Subjects underwent baPWV measurement after at least 5 min of rest in the supine position. Coffee, tea, cigarette use, or alcohol use were not allowed for 30 min before the test. Trained technicians and physicians placed pressure cuffs on both arms and ankles. The lower edge of the arm cuff was positioned 2–3 cm above the cubital fossa transverse striation, while the lower edge of the ankle cuff was positioned 1–2 cm above the medial malleolus. The heartbeat monitor was placed on the left edge of the sternum, and the electrocardiogram electrodes were placed immediately adjacent to it. Two bilateral readings of baPWV measurements were taken simultaneously, and the maximum readings of the right and left baPWV were used for the analysis. The ABI and heart rate (HR) were recorded automatically.

### Statistical analysis

Continuous variables have been presented as means ± standard deviation, and categorical variables have been expressed as proportions. Characteristics were compared between groups with significance tests, using the chi-squared test for categorical variables and one-way analysis of variance (ANOVA) for continuous variables. According to the 2019 ESC / EAS guidelines for the management of dyslipidemia [11], the lipid goals for the high-risk population were LDL-C < 1.81 mmol/L, non-HDL-C <  2.59 mmol/L, and apoB < 0.8 mmol/L. As there was no standard cut-off point for discordance, this study selected the mentioned target values as the cut-off points (i.e., LDL-C: 1.81 mmol/L, non-HDL-C: 2.59 mmol/L, apoB: 0.80 mmol/L) to define discordance, which was defined as LDL-C ≥ the cut-off point and non-HDL-C or apoB < the cut-off point or vice versa. Thus, participants were divided into four mutually exclusive concordance/discordance groups: low/low (LDL-C < the cut-off point and non-HDL-C or apoB <the cut-off point), low/high (LDL-C < the cut-off point and non-HDL-C or apoB ≥ the cut-off point), high/low (LDL-C ≥ the cut-off point and non-HDL-C or apoB < the cut-off point), and high/high (LDL-C ≥ the cut-off point and non-HDL-C or apoB ≥the cut-off point); the characteristics of the groups were analysed. The correlation between baPWV and LDL-C or non-HDL-C or apoB in the samples was performed using a Pearson analysis. All variables with *P* <  0.05 on univariate analysis or those considered clinically relevant (i.e., gender) were included in the logistic regression model to investigate the odds of arterial stiffness for each of the concordance/discordance groups, with the low/low group as the reference; arterial stiffness was defined by inclusion in the upper quartile of the baPWV. Statistical analysis was performed using the SPSS (version 22.0; SPSS Inc., Chicago, USA) software package, and a two-tailed *P* value < 0.05 was considered statistically significant.

## Results

The mean age of the study participants was 65.9 ± 13.0 years, and 59.5% of the participants were male. Overall, 77.6% had hypertension, 39.3% had diabetes, 39.8% were smokers, 25.6% were drinkers, and 44.8% were receiving lipid-lowering therapy (including statins, ezetimibe, and proprietary Chinese medicine). The mean LDL-C was 2.41 ± 0.81 mmol/L, non-HDL-C: 3.06 ± 0.94 mmol/L, apoB: 0.84 ± 0.21 mmol/L, and baPWV: 1712.9 ± 405.8 cm/s.

**Fig. 1:**
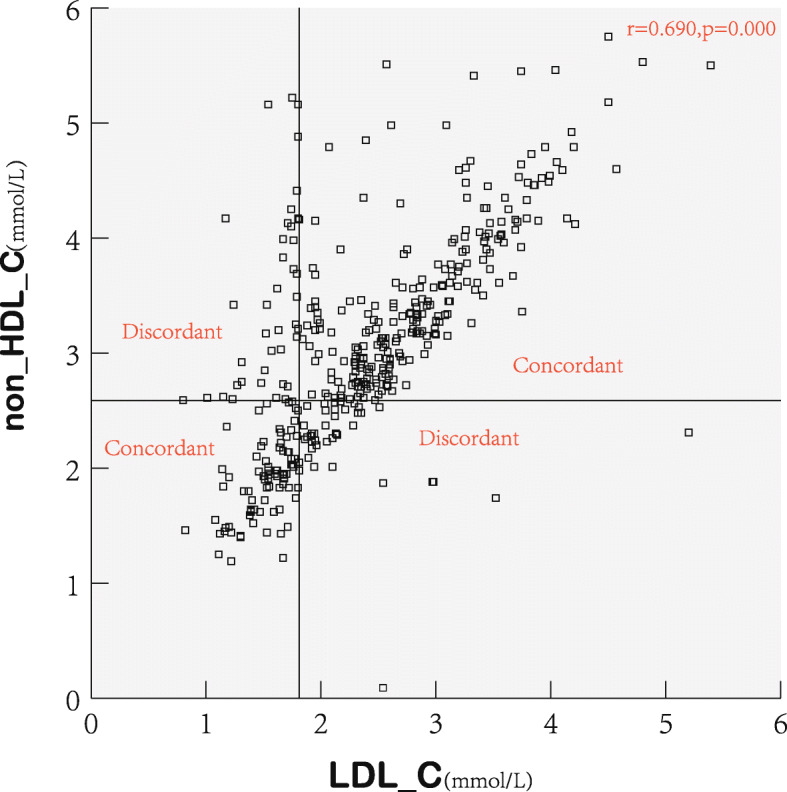
Scatterplots and prevalence of discordance and concordance based on cut-off points for LDL-C and non-HDL-C

**Fig. 2:**
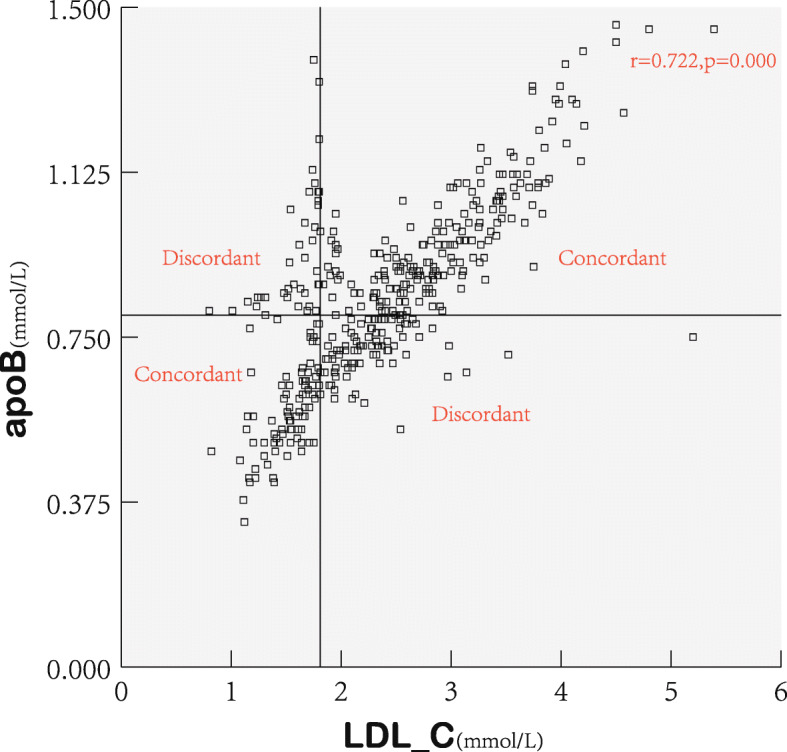
Scatterplots and prevalence of discordance and concordance based on cut-off points for LDL-C and apoB

LDL-C levels correlated positively with non-HDL-C and apoB levels (*r* = 0.690 and *r* = 0.722, respectively), but there was discordance between them (Figs. 1 and 2). There were 285 participants (70.9%) with LDL-C ≥ 1.81 mmol/L, 280 participants (69.7%) with non-HDL-C ≥ 2.59 mmol/L, and 235 participants (58.5%) with apoB≥0.80 mmol/L. Among the 285 participants with LDL-C ≥ 1.81 mmol/L, 43 (15.1%) had lower non-HDL-C, and 87 (30.5%) had lower apoB than the cut-off value. Among the 117 participants with LDL-C < 1.81 mmol/L, 38 (32.5%) had higher non-HDL-C, and 37 (31.6%) had higher apoB levels than the cut-off value. Overall, LDL-C was observed to be discordant with non-HDL-C (20.1%) and apoB (30.8%).

There were significant differences in the levels of SBP, DBP, LDL-C, non-HDL-C, TG, TC, apoB, baPWV, and the proportion of lipid-lowering drugs in the four concordance/discordance groups (Tables 1 and 2). In the participants with non-HDL-C or apoB levels higher than the cut-off value, the levels of SBP, DBP, non-HDL-C, TG, TC, apoB, and baPWV were all increased, while the proportion of lipid-lowering drugs was lower.

**Table 1:** Baseline characteristics of participants by LDL-C/Non-HDL-C concordance/discordance

Variables	Low/Low	Low/High	High/Low	High/High	*p*
N	79	38	43	242	
Age, year	65.1 ± 12.8	64.0 ± 15.1	65.6 ± 12.1	66.6 ± 12.9	0.596
Male, n (%)	52 (65.8)	22 (57.9)	28 (65.1)	137 (56.6)	0.432
Smoking, n (%)	32 (40.5)	14 (36.8)	17 (39.5)	97 (40.1)	0.983
Drinking, n (%)	27 (34.2)	9 (23.7)	14 (32.6)	53 (21.9)	0.114
Hypertension, n (%)	62 (78.5)	25 (65.8)	34 (79.1)	191 (78.9)	0.336
DM, n (%)	30 (38.0)	14 (36.8)	15 (34.9)	99 (40.9)	0.857
SBP, mmHg	122.7 ± 16.5	125.1 ± 16.4	124.8 ± 15.1	134.3 ± 17.8	0.000
DBP, mmHg	72.8 ± 10.9	72.1 ± 7.3	72.2 ± 8.1	76.6 ± 10.1	0.001
BMI, kg/m^2^	25.0 ± 3.2	25.6 ± 3.6	26.0 ± 2.7	25.7 ± 3.5	0.312
FBG, mmol/L	5.8 ± 2.3	5.9 ± 1.9	5.5 ± 1.5	6.0 ± 2.4	0.464
HDL-C, mmol/L	1.22 ± 0.35	1.09 ± 0.30	1.19 ± 0.31	1.17 ± 0.30	0.164
LDL-C, mmol/L	1.52 ± 0.22	1.58 ± 0.25	2.25 ± 0.63	2.85 ± 0.64	0.000^*^
Non-HDL-C, mmol/L	1.89 ± 0.34	3.50 ± 0.79	2.27 ± 0.49	3.51 ± 0.70	0.000^#^
TC, mmol/L	3.12 ± 0.47	4.59 ± 0.82	3.46 ± 0.58	4.68 ± 0.71	0.000
TG, mmol/L	1.34 ± 1.03	2.11 ± 2.18	1.19 ± 0.47	2.08 ± 1.56	0.000
apoB, mmol/L	0.58 ± 0.10	0.94 ± 0.15	0.71 ± 0.10	0.94 ± 0.16	0.000^#^
UA, μmol/L	324.8 ± 76.2	354.7 ± 76.5	350.3 ± 89.2	355.2 ± 89.6	0.055
homocysteine, mmol/L	14.4 ± 7.0	14.2 ± 9.2	14.0 ± 5.0	15.0 ± 8.1	0.812
eGFR, mL/(min·1.73 m^2^)	93.3 ± 16.3	94.5 ± 15.5	92.0 ± 14.7	89.6 ± 17.7	0.184
HGB, g/L	136.3 ± 14.7	135.1 ± 14.6	134.7 ± 13.3	136.7 ± 16.4	0.847
HR, bpm	67.6 ± 10.2	68.8 ± 9.2	65.4 ± 9.6	68.9 ± 9.5	0.148
Lipid-lowering therapy, n (%)	52 (65.8)	19 (50.0)	28 (65.1)	81 (33.5)	0.000
baPWV, cm/s	1533.0 ± 330.9	1713.3 ± 604.8	1605.4 ± 221.4	1790.7 ± 393.8	0.000

^*^The difference of LDL-C between the Low/Low group and the Low/High group was not statistically significant, and the difference between the other two groups was statistically significant (*P* = 0.000)

^#^ The difference of non-HDL-C or apoB between the High/Low group and the High/High group was not statistically significant, and the difference between the other two groups was statistically significant (*P* = 0.000)

**Table 2:** Baseline characteristics of participants by LDL-C/apoB concordance/discordance

Variables	Low/Low	Low/High	High/Low	High/High	*p*
N	80	37	87	198	
Age, year	64.7 ± 12.8	64.8 ± 15.1	66.5 ± 13.6	66.4 ± 12.4	0.684
Male, n (%)	52 (65.0)	22 (59.5)	50 (57.5)	115 (58.1)	0.725
Smoking, n (%)	32 (40.0)	14 (37.8)	32 (36.8)	82 (41.4)	0.895
Drinking, n (%)	27 (33.8)	9 (24.3)	24 (27.6)	43 (21.7)	0.206
Hypertension, n (%)	63 (78.8)	24 (64.9)	65 (74.7)	60 (80.8)	0.164
DM, n (%)	31 (38.8)	13 (35.1)	28 (32.2)	86 (43.4)	0.315
SBP, mmHg	122.3 ± 16.3	126.2 ± 16.7	128.9 ± 15.8	134.6 ± 18.3	0.000
DBP, mmHg	72.5 ± 10.6	72.8 ± 8.2	73.8 ± 9.8	76.9 ± 9.9	0.002
BMI, kg/m^2^	25.0 ± 3.1	25.5 ± 3.8	25.3 ± 3.1	25.9 ± 3.5	0.236
FBG, mmol/L	5.9 ± 2.4	5.8 ± 1.7	5.6 ± 1.8	6.1 ± 2.5	0.237
HDL-C, mmol/L	1.22 ± 0.35	1.19 ± 0.30	1.23 ± 0.34	1.15 ± 0.29	0.054
LDL-C, mmol/L	1.51 ± 0.22	1.59 ± 0.25	2.29 ± 0.45	2.97 ± 0.66	0.000^*^
Non-HDL-C, mmol/L	1.93 ± 0.44	3.46 ± 0.81	2.61 ± 0.39	3.64 ± 0.74	0.000^#^
TC, mmol/L	3.15 ± 0.51	4.56 ± 0.86	3.83 ± 0.48	4.79 ± 0.76	0.000
TG, mmol/L	1.48 ± 1.62	1.82 ± 1.34	1.49 ± 0.76	2.14 ± 1.67	0.000
apoB, mmol/L	0.58 ± 0.10	0.95 ± 0.15	0.72 ± 0.06	0.98 ± 0.15	0.000^#^
UA, μmol/L	343.6 ± 75.5	358.0 ± 76.7	346.8 ± 90.4	357.8 ± 89.0	0.062
homocysteine, mmol/L	14.3 ± 7.0	14.4 ± 9.3	14.7 ± 7.0	14.9 ± 8.0	0.946
eGFR, mL/(min·1.73 m^2^)	94.2 ± 15.6	92.7 ± 16.8	90.4 ± 16.4	89.8 ± 17.7	0.232
HGB, g/L	136.7 ± 14.0	135.3 ± 16.1	135.9 ± 15.4	136.4 ± 15.9	0.060
HR, bpm	67.4 ± 9.7	68.3 ± 10.1	66.9 ± 8.7	68.4 ± 9.7	0.064
Lipid-lowering therapy, n (%)	54 (67.5)	17 (45.9)	39 (44.8)	70 (35.4)	0.000
baPWV, cm/s	1525.7 ± 327.0	1733.9 ± 609.4	1684.0 ± 364.2	1797.3 ± 380.4	0.000

^*^The difference of LDL-C between the Low/Low group and the Low/High group was not statistically significant, and the difference between the other two groups was statistically significant (*P* = 0.000)

^#^The difference of non-HDL-C or apoB between the High/Low group and the High/High group was not statistically significant, and the difference between the other two groups was statistically significant (*P* = 0.000)

When stratified according to LDL-C levels, the baPWVs were greater in those with higher (≥ 2.59 mmol/L) compared to those with lower (< 2.59 mmol/L) non-HDL-C levels (LDL-C < 1.81 mmol/L:1713.3 ± 604.8 cm/s versus 1533.0 ± 330.9 cm/s, *P* = 0.039; LDL-C ≥ 1.81 mmol/L: 1790.7 ± 393.8 cm/s versus 1605.4 ± 221.4 cm/s, *P* = 0.000; Table 3). Similar trends were observed in those with higher (≥0.80 mmol/L) compared to those with lower (< 0.80 mmol/L) apoB levels (LDL-C < 1.81 mmol/L: 1733.9 ± 609.4 cm/s versus 1525.7 ± 327.0 cm/s, *P* = 0.018; LDL-C ≥ 1.81 mmol/L: 1797.3 ± 380.4 cm/s versus 1684.0 ± 364.2 cm/s, *P* = 0.020; Table 3)

**Table 3:** baPWV of participants by LDL-C/Non-HDL-C or apoB concordance/discordance

Groups		Non-HDL-C, mmol/L		ApoB, mmol/L	
		< 2.59	≥ 2.59	< 0.8	≥ 0.8
LDL < 1.81 mmol/L	n	79	38	80	37
	baPWV, cm/s	1533.0 ± 330.9	1713.3 ± 604.8	1525.7 ± 327.0	1733.9 ± 609.4
	F	2.953		5.781	
	*P*	0.039		0.018	
LDL ≥ 1.81 mmol/L	n	43	242	87	198
	baPWV, cm/s	1605.4 ± 221.4	1790.7 ± 393.8	1684.0 ± 364.2	1797.3 ± 380.4
	F	19.278		5.509	
	*P*	0.000		0.020	

Table 4 displays the odds ratios (ORs) of arterial stiffness for the four concordance/discordance groups of each set of LDL-C and non-HDL-C or apoB groups separately, after adjusting for age, sex, smoking, drinking, hypertension, diabetes, SBP, TG, eGFR, HGB, lipid-lowering therapy, BMI, and HR. The low/low groups constituted the reference in the logistic regression model. The ORs of the high LDL-C/low non-HDL-C and high LDL-C/low apoB groups were not significantly higher than those of the reference. In contrast, the ORs of the low LDL-C/high non-HDL-C and low LDL-C/high apoB groups were significantly higher than those of the reference. The high LDL-C/high non-HDL-C and high LDL-C/high apoB groups were also significantly higher than those in the reference.

**Table 4:** Concordance/Discordance between LDL-C and Non-HDL-C or apoB groups in relation to arterial stiffness risk

LDL-C/Non-HDL-C	OR (95% CI)	*P*	LDL-C/apoB	OR (95% CI)	*P*
Low/Low (referent)	1	0.001	Low/low (referent)	1	0.005
Low/High	13.412 (2.341–76.850)	0.004	Low/high	13.054 (2.385–71.454)	0.003
High/Low	0.268 (0.044–1.618)	0.151	High/low	1.214 (0.408–3.609)	0.727
High/High	3.174 (1.219–8.262)	0.018	High/high	3.062 (1.147–8.179)	0.026

## Discussion

This study was conducted in a population of middle-aged and elderly Chinese individuals, who are known to have a high prevalence of arterial stiffness. The lipid goal for the high-risk population according to the 2019 ESC/EAS Guidelines was selected as the cut-off point in this study. In this cohort, LDL-C was discordant with non-HDL-C (20.1%) and apoB (30.8%); the values were higher than the results reported by Lawler et al. [20] and Wilkins et al. [21]. Lower non-HDL-C and apoB levels were associated with lower baPWV, whereas higher non-HDL-C and apoB levels were associated with higher baPWV. In the discordant groups, the odds for arterial stiffness were significantly higher when non-HDL-C or apoB was greater than the cut-off value, and not significantly higher than the reference when non-HDL-C or apoB levels were below the cut-off point, suggesting that the risk for arterial stiffness is more strongly influenced by non-HDL-C or apoB than by LDL-C. Notably, participants with high LDL-C and high non-HDL-C or apoB also had high risk for arterial stiffness; the particles were numerous, but of an average cholesterol concentration. These results suggested that only patients with non-HDL-C or apoB levels higher than the cut-off value had an increased risk of arterial stiffness; thus, the increased risk of arterial stiffness may be due to the significant differences in non-HDL-C or apoB between the groups. In addition, there was a trend towards high arterial stiffness risk in the discordant groups with low non-HDL-C or apoB and high LDL-C, possibly because their non-HDL-C and apoB increased by 0.38 mmol/L and 0.14 mmol/L, respectively, compared with the reference.

Arterial stiffness measured by baPWV is considered a marker of subclinical atherosclerosis, and an independent risk predictor of CVD [22]. Therefore, the identification of serum biomarkers associated with arterial stiffness will provide considerable advantages in preventing atherosclerosis and CVD, and confer substantial clinical benefits. In the case of similar blood pressure and age, arterial stiffness was more serious in patients with hypercholesterolemia than that in those with normal blood lipid levels [23]. In addition, several clinical trials have shown that lipid-lowering therapy can improve the baPWV [24]. Thus, there was a correlation between dyslipidemia and arterial stiffness. LDL-C has long been the major target of lipid-lowering therapies, while non-HDL-C and apoB are still controversial targets. Nevertheless, many studies have confirmed the effect of non-HDL-C or apoB on arterial stiffness. Furthermore, a Dutch study involving 1517 participants supported the use of non-HDL-C as a superior predictor of LDL-C in identifying individuals with arterial stiffness [25]. A recent meta-analysis by Upala et al. including 303 participants [26], also reported an association between statin therapy and PWV in the lower aortic segment. In several studies from China [27, 28], non-HDL-C was more strongly associated with baPWV than other lipid parameters. This correlation was significant in both men and women, suggesting that non-HDL-C was a surrogate lipid marker of the arterial stiffness level. In a study on patients with familial hypobetalipoproteinemia (FHBL) [29], an attenuated gradual increase in arterial stiffness was found; lowering of apoB-containing lipoproteins should therefore have beneficial impact on the vascular system in subjects with “non-cholesterol” risk factors. The Nijmegen Biomedical Study had shown that an elevated apoB level was a marker of more severer arterial stiffness [30]. Studies from South Korea [31] and Finland [32] showed that elevations of apoB or non-HDL-Care associated with increased arterial stiffness in young adults, and an increase in apoB could lead to an increase in arterial stiffness. A study on adolescents with type 1 diabetes had shown that elevated apoB was significantly associated with increased arterial stiffness, especially in those with borderline LDL-C (2.59–3.34 mmol/L), and apoB in addition to LDL-C might help stratify the CVD risk [33].

### Study strength and limitations

Discordance analysis was helpful in identifying the “residual risk; this is a relatively new approach to epidemiological analysis. The most clinically relevant question appears to be whether discordance relates to greater CVD in those who have discordance. Related lipid parameters should therefore be compared for risk signals when they disagree, not when they agree. The strength of this study is that the proportion of discordance in lipid parameters was sizable; this will facilitate studies on whether discordance in lipid parameters has an effect on the relationship between arterial stiffness and blood lipids. This study had several limitations. First, the cross-sectional design may have introduced selection bias, and the sample size was small; therefore, participants in this study may not represent the general middle-aged and elderly population. Second, nearly half of the participants in the study were receiving lipid-lowering therapy; this could affect the relationship between blood lipids and arterial stiffness. Finally, there is no absolute definition and no standard cut-off point for discordance between LDL-C, non-HDL-C, and apoB; therefore, changing the definition and cut-off points could affect the results.

## Conclusions

In conclusion, non-HDL-C or apoB is discordant with LDL-C in middle-aged and elderly Chinese individuals; this may significantly affect arterial stiffness. Lipid-lowering therapy in these individuals should therefore not only focus on LDL-C levels, but also on non-HDL-C and apoB levels to further reduce arterial stiffness. When discordant with LDL-C, non-HDL-C or apoB may identify individuals who may benefit from more comprehensive lipid modification. It is hoped that new lipid-lowering drugs in the future will target the discordance in lipid parameters.

## Data Availability

The datasets used and/or analysed during the current study are available from the corresponding author on reasonable request.

## Abbreviations

apoB: Apolipoprotein B
AS: Arterial stiffness
AST: Aspartate aminotransferase
ALT: Alanine aminotransferase
ABI: Ankle/brachial systolic blood pressure index
baPWV: Brachial-ankle pulse wave conduction velocity
BMI: Body mass index
CVD: Cardiovascular disease
CKD-EPI: Chronic kidney disease epidemiology collaboration
DBP: Diastolic blood pressure
DM: Diabetes mellitus
eGFR: Estimated glomerular filtration rate
ESC / EAS: European society of cardiology / european atherosclerosis society
FBG: Fasting blood glucose
FHBL: Familial hypobetalipoproteinemia
HbA1C: Glycosylated haemoglobin
HDL-C: High-density lipoprotein cholesterol
HGB: Haemoglobin
HR: Heart rate
LDL-C: Low-density lipoprotein cholesterol
LPa: Lipoprotein a
non-HDL-C: Non-high-density lipoprotein cholesterol
NHANES: National health and nutrition examination survey
ANOVA: One-way analysis of variance
Scr: Serum creatinine
SBP: Systolic blood pressure
TC: Total cholesterol
TG: Triglyceride
UA: Uric acid
